# Mesenchymal chondrosarcoma of the right buccal region: A case report and review of the literature

**DOI:** 10.3892/ol.2014.2595

**Published:** 2014-10-09

**Authors:** LIJIANG YU, MINGLIANG LI, RUNTAI LIN, YUE MU, JIZHI ZHAO

**Affiliations:** Department of Oral and Maxillofacial Surgery, Peking Union Medical College Hospital, Chinese Academy of Medical Sciences, Beijing 100730, P.R. China

**Keywords:** mesenchymal chondrosarcoma, soft-tissue neoplasms, computed tomography, calcification

## Abstract

Extraskeletal mesenchymal chondrosarcoma (EMCS) is a rare malignant cartilaginous tumor arising from the soft tissues. The most common areas of extraskeletal origin are the lower extremities, the orbits and the central nervous system, among others. In this study, the case of primary EMCS arising from the right buccal region in a 26-year old female is presented. Histological and immunohistochemical analysis confirmed the diagnosis of EMCS. Subseqently, the patient was treated with radical surgery, but declined chemotherapy or radiotherapy, which was recommended. One year after surgery, no recurrence had been identified in the patient. To the best of our knowledge, only one case of primary EMCS of the buccal region has been reported previously. In the current study, a case of primary EMCS of the buccal region is presented.

## Introduction

Mesenchymal chondrosarcoma (MCS) is a rare and highly aggressive pathological variant of CS arising from the soft and hard tissues (1). MCS accounts for 3–9% of all CS cases (2–7) and was first identified by Lichtenstein and Bernstein (6) in 1959. In total, 30–50% of MCSs are extraskeletal in origin. Extraskeletal MCS (EMCS) usually occurs in the second and third decades of life (7) and affects females more frequently than males (8). The neoplasm is rare and aggressive, with a high likelihood for delayed distant metastasis and late recurrence (3,9), and with a poor prognosis and survival rate (10). The most common sites of extraskeletal origin (11–13) include the lower extremities, orbits and central nervous system, among others. Only 6% of EMCSs arise from soft tissue of the head and neck region (14). Histopathological examination of EMCS reveals a tumor composed of atypical undifferentiated small cells and islands of matured chondroid matrix, typically with a bimorphic appearance. Computed tomography scans usually reveal the presence of various patterns of calcification within the lesion.

To the best of our knowledge, only one previous case of primary EMCS involving the buccal region has been reported. In the current study, a novel case of primary EMCS arising from the right buccal region in a 26-year-old female is presented and the literature is reviewed, with a focus on the management of this tumor. The patient provided written informed consent.

## Case report

**Figure 1:**
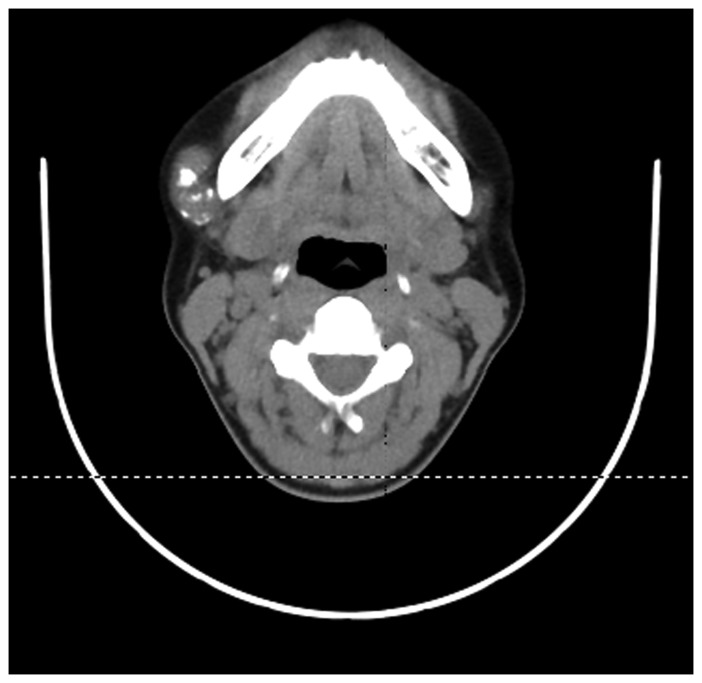
Computed tomography scan revealing a soft-tissue mass with dense calcification located in the right buccal region.

**Figure 2:**
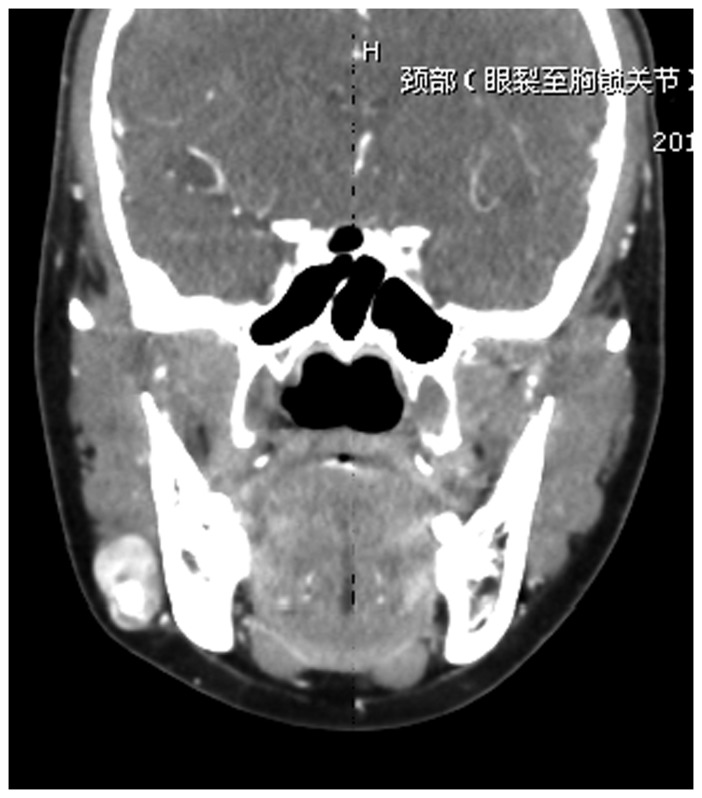
Diffuse heterogeneous enhancement was visible following an enhanced scan and the calcified area had also been strengthened.

**Figure 3:**
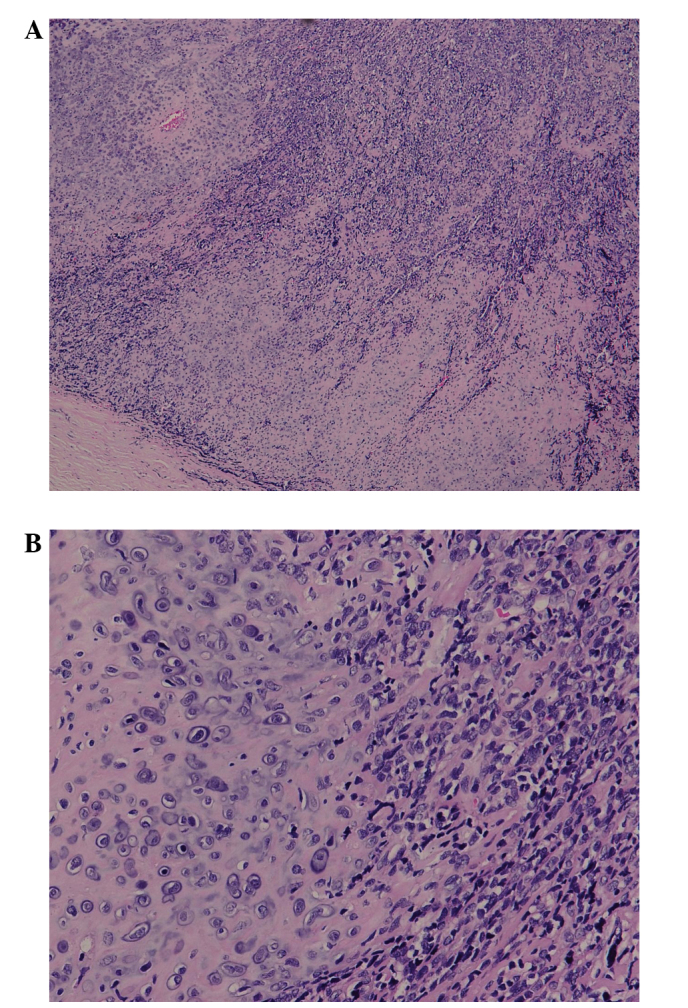
Pathological specimens revealing (A) a hemangioperictyoma-like vascular pattern, with proliferation of small, round and undifferentiated mesenchymal cells with clear cytoplasm and (B) small round cells surrounding the blood vessels and enveloping the differentiated cartilage (stain, hematoxylin and eosin; magnification, ×200).

A 26-year-old female, with no medical history, presented to the surgical Out-patients Department of the Oral and Maxillofacial Surgery of Peking University Medical College Hospital (Chinese Academy of Medical Sciences, Beijing, China), with the chief complaint of a mass in the right buccal region. The mass was painless and had gradually increased in size for 12 months. Six months previously, upon investigation at another hospital, apparent upper mandibular lymph node swelling was detected in the right buccal region. The patient was provisionally diagnosed with lymph node inflammation and was treated with antibiotics. However, the patient’s condition deteriorated two weeks prior to the presentation to the Peking University Medical College Hospital. Extraoral examination revealed a firm mass measuring ~3×2.5 cm in size, without fixation to the mandible. The overlying skin color and texture was normal. The mass was well defined and lobulated on palpation. The facial nerve was not involved with the tumor. A physical examination and chest X-ray revealed no clinical evidence of distant metastasis. Computed tomography (CT) scans revealed a well-defined mass, without involvement of the mandible (Figs. 1 and 2). The mass was widely resected under general anesthesia and was subsequently histopathologically investigated. The post-operative course was uneventful. The gross appearance of the mass was typically grey or tan in color and poorly circumscribed. Microscopy revealed a tumor composed of islands of well-differentiated cartilage surrounded by areas of ovoid- and spindle-shaped cells exhibiting a hemangiopericytomatous pattern (Figs. 3 and 4). Focal calcification was observed in chondroid areas. Immunohistochemical analysis of the tumor cells revealed positivity for vimentin, S-100, AE1/AE3, B-cell lymphoma-2, cluster of differentiation (CD)99 and leukocyte common antigen, and negativity for desmin, epithelial membrane antigen (EMA) and CD34. Histology and immunohistochemistry indicated the diagnosis of EMCS. The patient declined chemotherapy and radiotherapy, but continued to attend follow-up appointments. No local recurrence or metastasis was identified following surgery. To date, no local reccurence or metastasis has been identified in the patient.

## Discussion

MCS is a rare and highly aggressive pathological variant of CS arising from the soft and hard tissues. MCS accounts for 3–9% of all CSs. In total, 30–50% of MCSs are extraskeletal in origin. The most common sites of extraskeletal origin are the lower extremities, orbits and central nervous system, among others. Rare occurrences have also been identified in the lungs, ribs, pleura, spleen and kidneys. EMCS accounts for only 2% of all soft-tissue sarcomas, and only 6% arise from the soft tissue of the head and neck region. EMCS exhibits two peaks of incidence in adults, depending on its location. EMCS of the head and neck usually affects patients in the second or third decades of life, whereas EMCS of the deep muscles usually affects patients in the fifth decade of life (3,14,15). EMCS exerts a slight predominance in females. In individuals >30 years old, the disease more frequently affects the trunk or the soft tissues of the limbs. At present, only one previous case of primary EMCS involving the buccal region has been reported. To the best of our knowledge, the present study is the second case of primary EMCS arising from the right buccal region to be reported.

The clinical signs and symptoms of EMCS include pain and the presence of a soft mass. A variety of signs and symptoms are associated with the primary site of involvement, including facial paresthesia and lip paresis. In certain cases, local surgery or biopsy may lead to rapid growth. EMSCs are also occasionally misdiagnosed. The main differential diagnoses of EMCS include rhabdomyosarcoma, Ewing’s sarcoma, hemangiopericytoma, synovial sarcoma and other variants of CS, as these also exhibit small round blue cells (14,16), particularly in biopsy specimens and fine-needle aspiration cytology (FNAC). Therefore, it is hypothesized that a pre-operative FNAC pathological examination is indicative for the correct therapy. However, researchers from Modena University reported a case of pleural MCS that was incorrectly diagnosed by FNAC (17). Therefore, the diagnosis of EMCS on the basis of FNAC remains controversial. This is due to the difficulty in sampling the two phases of the tumor, the lack of distinctive clinical and radiographical features and the rarity of the tumor. Buchon *et al* (18) suggested wide sampling of the tumor mass, to detect the cartilaginous component, which is diagnostic of EMCS.

Typical histological features of MCS, consisting of small, round or spindled mesenchymal cells interspersed with islands of hyaline cartilage, have been identified (10,19). Immunohistochemistry is important, particularly in small biopsies. Typical immunohistochemistry results include the positivity of the mesenchymal portion for vimentin, Leu-7 and CD99 (20), and the positivity of the cartilaginous areas for S-100 protein. However, S-100 positivity is not specific for CSs and may be observed in other tumors, including melanomas, malignant peripheral nerve sheath tumors, schwannomas and histiocytomas. Immunonegativity for cytokeratin, EMA and CD34 is observed in EMCS (21,22). Focal positivity for cytokeratin and EMA is usually exhibitedby Synovial sarcomas, while hemangiopericytomas exhibit immunopositivity for CD34 and EMA. Hoang *et al* (23) found Sox9 to be consistently positive in the cartilaginous and primitive mesenchymal cells in EMCS, thus aiding in the differentiation from other small round blue cell tumors. Collagen type II (24) and friend leukemia virus integration-1 (25) may prove to be diagnostic tools, even in MCS samples lacking cartilage islands.

Although the radiographical appearance is generally not specific, the two component structures of EMCS with differentiated cartilage islands interspersed within vascular undifferentiated mesenchyme are also frequently observed in EMCS (2,26). Previously, it was reported that 50–100% of EMCSs demonstrate calcification on plain radiography and CT scans (15,27,28). The uncalcified areas exhibit low attenuation similar to muscle on CT. T2-weighted magnetic resonance imaging (MRI) clearly show a two-component structure composed of calcified and uncalcified areas (4,25,26), and enhanced MRI reveals heterogeneous enhancement of each area.

At present, early surgery is the standard local treatment, with studies demonstrating improved survival in patients who undergo wide surgical resection (29). Zakkak *et al* (30) reviewed various treatment modalities in the maxilla and mandible and found that the best outcome was observed following radical surgery. Radiotherapy may be important (31), however, certain studies have indicated that EMCS is a radioresistant tumor (32–35). Previous studies have reported the use of pre-operative radiation therapy to reduce the tumor bulk prior to radical resection and prevent further extension and micrometastasis. However, this does not affect the pre-operative approach. No improvement in prognosis has been identified with post-operative radiotherapy, even when there is evidence demonstrating a trend toward increased survival. Chemotherapy plays a limited role and thus, should be used for high-grade mesenchymal tumors, local recurrence with aggressive behavior or in cases with potential for metastasis. A study of 35 cases demonstrated that poorly-differentiated MCSs responds to combined chemotherapy and radiotherapy, while combined neoadjuvant chemotherapy and surgery are more effective against more differentiated tumors, such as the hemangiopericytoma-like variant (32). Cesari *et al* (29) demonstrated clear results supporting the use of chemotherapy, whereby the disease-free survival rate of patients at 5–10 years after surgical remission of the disease was 76 and 17% with and without chemotherapy, respectively (29). However, two additional studies contradict these results with regard to chemotherapy and radiotherapy, reporting that only radical surgery significantly improves survival, while radiotherapy and chemotherapy may be of palliative use, independent of the age of the patient, the site of the tumor and the histological variant (36,37).

The prognosis is extremely variable, with published five-year survival rates ranging between 42 and 80.7% (38,39) and 10-year survival rates ranging between 21 and 67% (3,13,29,41,42).

In the present study, a rare case of primary EMCS in the buccal region, with aggressive clinical, radiographic and histological features was presented. Early radical removal of the tumor is the standard treatment for EMCS. Adjuvant radiation therapy and chemotherapy must be considered, due to the histologically aggressive nature of the tumor. EMCS is a rare entity that must be considered in the differential diagnosis of soft-tissue neoplasms with calcification in the oral and maxillofacial region, particularly in young adults.
